# The dense-core plaques of Alzheimer’s disease are granulomas

**DOI:** 10.1084/jem.20212477

**Published:** 2022-06-22

**Authors:** Greg Lemke, Youtong Huang

**Affiliations:** 1 Molecular Neurobiology Laboratory, The Salk Institute for Biological Studies, La Jolla, CA; 2 Immunobiology and Microbial Pathogenesis Laboratory, The Salk Institute for Biological Studies, La Jolla, CA

## Abstract

Dense-core plaques, whose centers contain highly polymerized and compacted aggregates of amyloid β peptides, are one of the two defining histopathological features of Alzheimer’s disease. Recent findings indicate that these plaques do not form spontaneously but are instead constructed by microglia, the tissue macrophages of the central nervous system. We discuss cellular, structural, functional, and gene expression criteria by which the microglial assembly of dense-core plaques in the Alzheimer’s brain parallels the construction of granulomas by macrophages in other settings. We compare the genesis of these plaques to the macrophage assembly of mycobacterial granulomas, the defining histopathological features of tuberculosis. We suggest that if dense-core plaques are indeed granulomas, their simple disassembly may be contraindicated as an Alzheimer’s therapy.

## Teaser

Microglia, the tissue macrophages of the central nervous system, use phagocytosis of loosely organized amyloid beta (Aβ) to construct dense-core plaques, one of the defining histopathological features of Alzheimer’s disease. The biology of this process parallels macrophage construction of granulomas in tuberculosis and other settings.

## Introduction

Granulomas are compact, organized collections of mononuclear phagocytes—primarily macrophages (Adams, 1976)—that develop in response to an unresolved infectious or foreign body stimulus (Anderson et al., 2008; Pagan and Ramakrishnan, 2018; Williams and Williams, 1983). These structures are common, and are seen in schistosomiasis (Takaki et al., 2021), after the inhalation of silica and metals (Leung et al., 2012), in atherosclerosis (Johnson and Newby, 2009), and following the deposition of foreign bodies (Molina-Ruiz and Requena, 2015) or insoluble proteins or lipids (Terziroli Beretta-Piccoli et al., 2018). The most widely studied granulomas have been those that develop in tuberculosis (TB) and other persistent mycobacterial infections (Cadena et al., 2017; Davis and Ramakrishnan, 2009; Pagan and Ramakrishnan, 2014). TB is marked by the presence of pulmonary and extrapulmonary granulomas that are populated by macrophages, macrophage derivatives, and a panoply of other immune cells. Although some TB granulomas are paucibacillary, these cells typically surround a community of *Mycobacterium tuberculosis*. Traditionally, the TB granuloma has been viewed as a confinement structure that sequesters the bacillus (Williams and Williams, 1983). This view has been challenged, however, since in addition to protecting the host from the bacterium, this granuloma also provides an environment that enables bacterial growth and dissemination (Cambier et al., 2014; Ramakrishnan, 2012).

In this Perspective, we suggest that the dense-core amyloid β (Aβ) plaques of Alzheimer’s disease (AD), which were first described by Alois Alzheimer more than a century ago (Alzheimer et al., 1995), are granulomas. We compare the construction of these plaques to the development of the TB granuloma. Understanding dense-core plaque formation is important, since together with neurofibrillary tangles of the microtubule protein Tau, plaques are the defining histopathological feature of AD (DeTure and Dickson, 2019).

Historically, dense-core Aβ plaques have been identified through staining of postmortem brain sections with Congo Red, Thioflavin S or T (Thio S/T), and other dyes that insert into the β-pleated sheets of aggregated amyloid proteins (Klunk et al., 2002; Lee, 2002). They are now also detected in AD patients by positron emission tomography (PET), using radioactive analogs of Thio T (Klunk et al., 2004), florbetapir (Zeng and Goodman, 2013), and related compounds (Kepe et al., 2013). The protein at the center of dense-core plaques consists of compacted fibrillar polymers of Aβ peptides (Glenner et al., 1984; Masters et al., 1985). These peptides are generated by sequential proteolytic cleavage from the extracellular domain of the amyloid precursor protein (APP), a transmembrane receptor expressed by neurons and other cells (Selkoe, 2001). Although there is considerable diversity in the length and cleavage sites of Aβ peptides, most are 40–42 amino acids in length (O’Brien and Wong, 2011; Selkoe and Hardy, 2016). Aβ peptides multimerize in the extracellular space of the brain to form oligomers and proto-fibrils (Fontana et al., 2020; Thal et al., 2015). These polymers, most of which are potently toxic to neurons when assayed in vitro (Pike et al., 1993; Sakono and Zako, 2010), assemble into diffuse plaques that can be visualized using antibodies that recognize Aβ peptides (Baghallab et al., 2018; DeTure and Dickson, 2019; Huang et al., 2021). Diffuse plaques are amorphous deposits (Serrano-Pozo et al., 2011) that are not typically associated with the damaged neurites that mark dense-core plaques (Ringman et al., 2014). Importantly, they are not stained (for Congo Red) or only weakly stained (for Thio S) by the dyes that bind tightly to dense-core plaques (Condello et al., 2015; DeTure and Dickson, 2019).

Recent studies in the mouse indicate that dense-core plaques do not form spontaneously (Baik et al., 2016; Huang et al., 2021; Spangenberg et al., 2019). Instead, they are built by microglia, the most abundant tissue-resident macrophages of the central nervous system (CNS; Li and Barres, 2018; Prinz et al., 2021). Moreover, dense-core plaque formation appears to be dependent on microglial phagocytic activity (Huang et al., 2021). Microglia are now at the center of AD research, since many genetic polymorphisms that increase AD risk occur in genes that are, in the CNS, predominantly or exclusively expressed by these macrophages (Jansen et al., 2019; Long and Holtzman, 2019). Nevertheless, the centrality of microglia to dense-core plaque formation is not widely appreciated.

## Microglial association with dense-core plaques

**Figure 1.**
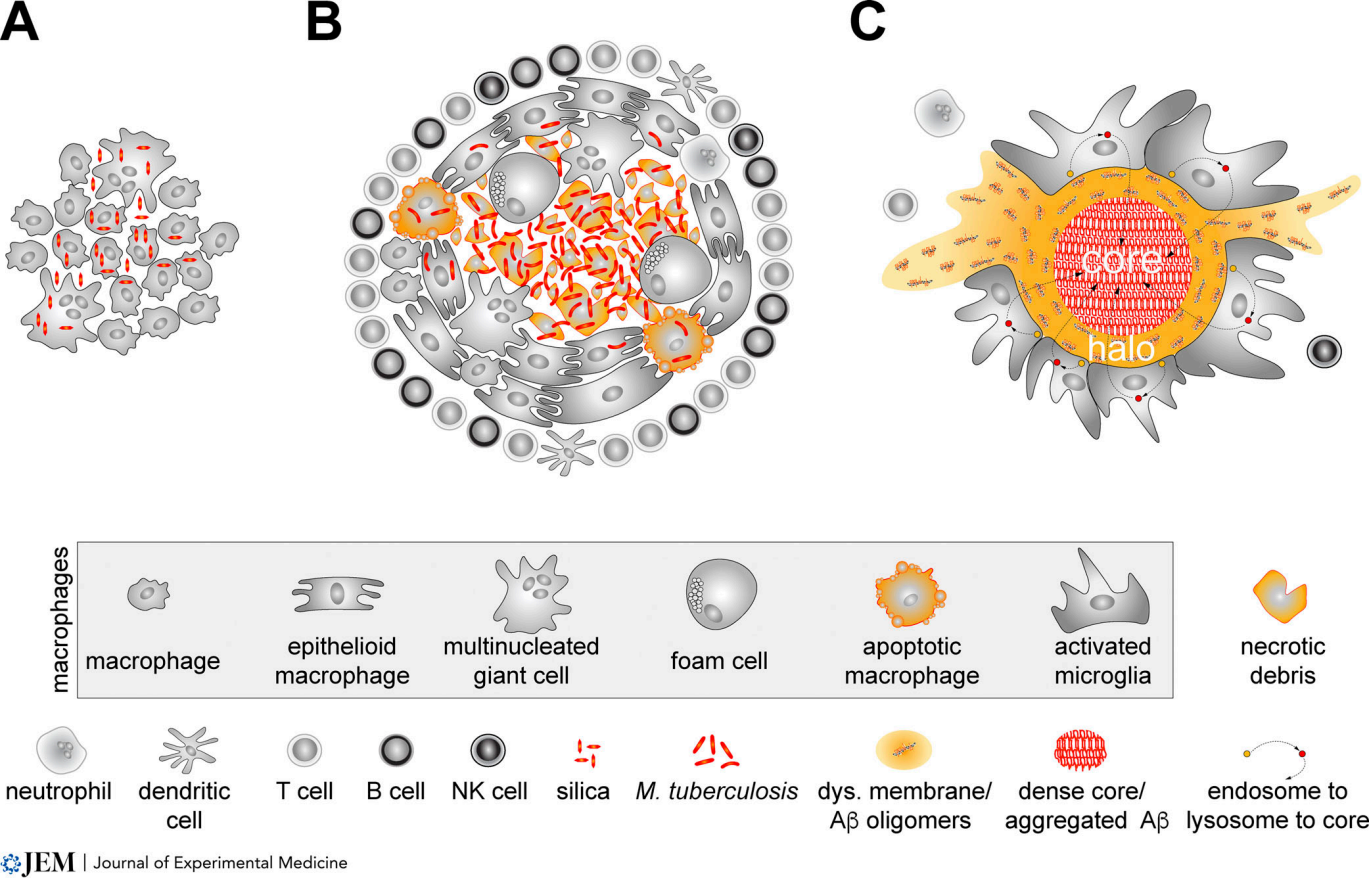
**Cellular configuration of three different granulomas. (A)** The simple foreign body granuloma surrounding inhaled silica crystals in the lung (red; seen in silicosis) comprises phagocytic macrophages and multinucleated giant cells. **(B)** The elaborate TB granuloma is comprised of activated macrophages and macrophage derivatives that include interdigitated epithelioid macrophages. (The latter are interlinked.) Together with multinucleated giant cells and foam cells, these macrophages may be infected by *M. tuberculosis* (red). Phagocytosis of *M. tuberculosis* leads to the apoptotic death of many macrophages. The center of the TB granuloma usually contains necrotic cells and debris, while its perimeter is ringed with the indicated cells of the innate and adaptive immune systems. **(C)** The dense-core plaque of AD is closely attended by activated microglia. The core of the plaque is composed of highly polymerized and compacted Aβ material (red). This dense core is surrounded by a halo of loosely organized, noncompacted Aβ oligomers and proto-fibrils (yellow) that are associated with PtdSer-displaying dystrophic membrane derived from cells damaged or killed by Aβ exposure. (Noncompacted Aβ oligomers and proto-fibrils also comprise diffuse plaques that lack a dense core.) As detailed in Fig. 2, halo material is converted to dense-core material via microglial phagocytosis (yellow and red circles, respectively). Small numbers of T cells, neutrophils, and NK cells have been reported in the AD brain, although these may be at some distance from Aβ plaques.

The triggering irritant of any granuloma is always surrounded by macrophages (Fig. 1). Many foreign body granulomas, such as those seen in silicosis (Fig. 1 A), are simple assemblies of macrophages and their derivatives (Leung et al., 2012). In contrast, the complex granulomas that develop in TB contain macrophages that undergo a series of specialized transformations to yield interdigitated epithelioid macrophages, foam cells, and giant cells (Pagan and Ramakrishnan, 2014; Pagan and Ramakrishnan, 2018). In addition to these cells, which are frequently arrayed around and infected by *M. tuberculosis*, TB granulomas are also populated by dendritic cells, neutrophils, natural killer (NK) cells, B cells, and CD4^+^ and CD8^+^ T cells (Cooper, 2009; Flynn et al., 1992; Ramakrishnan, 2012; Fig. 1 B). Although there is wide diversity in the structure of pulmonary TB granulomas, the centers of these granulomas are usually necrotic.

The association of microglia with dense-core plaques has been studied in both AD and its many amyloidogenic mouse models (https://www.alzforum.org/research-models/alzheimers-disease). These mouse models express mutant forms of the human *APP* gene that result in familial (inherited) AD. Often the *APP* genes are coexpressed with mutant forms of the human *Presenilin-1* (*PSEN1*) gene, which encodes a component of the enzyme complex that cleaves Aβ peptides from APP (Myers and McGonigle, 2019). These *PSEN1* mutations also result in familial AD. Most amyloidogenic AD mice overproduce Aβ, develop dense-core plaques, and eventually display overt disease. The association of microglia with Aβ plaques in these mice is not as elaborate as that seen for macrophages in the TB granuloma and more closely resembles the macrophage assemblies of foreign body granulomas (Fig. 1, A and C). Nonetheless, imaging studies in mouse AD models indicate that dense-core plaques are tightly enveloped by microglial cell bodies and their processes, with 50–60% of the plaque surface being covered by microglia on average at steady state (Baik et al., 2016; Condello et al., 2015; Spangenberg et al., 2019; Fig. 1 C). Some plaques in AD mouse brains are completely encapsulated by microglia. Although microglial process motility (Nimmerjahn, 2012; Nimmerjahn et al., 2005) is reduced in AD mouse brains (Huang et al., 2021), these processes may nonetheless palpate the entirety of the plaque surface several times each day.

Plaque-associated microglia (PAM) are transcriptionally and functionally altered relative to their non–plaque-associated counterparts (NPAM). They transform from an exploratory homeostatic phenotype characterized by the presence of many long, highly branched, and highly motile cell processes, to an activated amoeboid phenotype characterized by elevated expression of inflammatory cytokines and phagocytic mediators, swollen cell bodies, and fewer, shorter, and less motile processes (Huang et al., 2021; Keren-Shaul et al., 2017; Paasila et al., 2019; Roy et al., 2020; Yin et al., 2017; Zhou et al., 2020). Immediately peripheral to PAM are activated astrocytes (Rodriguez et al., 2009). T cells, NK cells, and neutrophils are more prominent in Aβ plaque–burdened brains than in healthy mouse brains, but these cells are not consistently seen in close association with plaques (Wyatt-Johnson and Brutkiewicz, 2020; Fig. 1 C). There is evidence that these additional immune cells, most notably CD4^+^ T cells, regulate neuroinflammation in AD (Gonzalez and Pacheco, 2014; Marsh et al., 2016). Consistent with this role, multiple mRNAs encoding proteins involved in antigen presentation are up-regulated specifically in PAM in the APP/PS1 mouse AD model (Huang et al., 2021).

## Plaque dependence on microglia

If dense-core plaques are granulomas, and all granulomas are built by macrophages, then dense-core plaques should never form in the absence of microglia. Investigators have addressed this question using pharmacological inhibition of a microglial survival pathway in mouse AD models before plaque formation. Genetic studies demonstrate that microglia and other tissue macrophages require continuous signaling through the colony-stimulating factor 1 receptor (CSF1R) to survive (Elmore et al., 2014; Otero et al., 2009), and in vivo blockade of this receptor tyrosine kinase (RTK), achieved via oral gavage or dietary delivery of small-molecule CSF1R kinase inhibitors, results in the apoptotic death and depletion of nearly all microglia from the mouse brain (Spangenberg et al., 2019; Szalay et al., 2016).

This depletion has been performed in the 5xFAD model (Oakley et al., 2006). Mice were administered a CSF1R inhibitor beginning at 1.5 mo of age, before the appearance of any plaques, and were analyzed for Thio S^+^ dense-core plaques in the cortex after 10 wk of treatment (Spangenberg et al., 2019). Almost all microglia were killed, and correspondingly, there was a dramatic reduction in the number of Thio S^+^ plaques relative to controls. Importantly, the few plaques remaining after treatment were seen to be in perfect correspondence with microglia that had escaped killing (Spangenberg et al., 2019). Coincident with the death of microglia, these 5xFAD mice developed vascular amyloid deposits, a well-described comorbidity in AD known as cerebral amyloid angiopathy (Greenberg et al., 2020). As seen in studies performed in other AD mouse models (Dagher et al., 2015; Olmos-Alonso et al., 2016), CSF1R inhibition had no effect on overall Aβ production.

An earlier analysis (Grathwohl et al., 2009) crossed a CD11b-herpes simplex virus thymidine kinase mouse line with an early-onset APP/PS1-21 mouse AD model (Radde et al., 2006). CD11b^+^ microglia were killed using ganciclovir, which is converted to a toxic triphosphate by the action of herpes simplex virus thymidine kinase and downstream kinases. After treatment, many Congo Red^+^ plaques were observed in brain regions where nearly all microglia had been killed, and so the study’s authors concluded that plaque formation was microglia independent (Grathwohl et al., 2009). The issue with these experiments is that ganciclovir treatment was initiated at a time when plaques were already being rapidly deposited, and analyses of microglial depletion and plaque density were performed only at the end of the experiment. Ganciclovir killing is relatively slow in many cells (Beck et al., 1995), and so microglia may have been present for much of the treatment period. More importantly, microglial depletion has been shown to have little or no effect on the stability of dense-core plaques once these plaques have already been deposited (Casali et al., 2020; Spangenberg et al., 2019). Thus, the preponderance of evidence argues that dense-core plaques are not formed in the absence of microglia.

## Microglial phagocytosis of Aβ

Granulomas are built by macrophage phagocytosis. Formation of the TB granuloma is dependent on both macrophage phagocytosis of *M. tuberculosis* and the ability of the bacillus to inhibit its destruction by phagocytosis (Davis and Ramakrishnan, 2009; Ramakrishnan, 2012). Indeed, all pathogens that trigger granulomas must evolve mechanisms to avoid phagocytic elimination. In TB, bacteria are engulfed by interstitial lung macrophages, having been transported into the lung parenchyma by alveolar macrophages (Cambier et al., 2014; Cohen et al., 2018; Huang et al., 2018). After internalization into macrophage endosomes, *M. tuberculosis* avoids lysosomal destruction by blocking endosome acidification and halting phagolysosome fusion (Vergne et al., 2004). Bacteria eventually populate the cytoplasm and kill the macrophage. Through reuptake of bacteria-laden necrotic debris and repeated transit through this cycle, *M. tuberculosis* uses macrophage phagocytosis to form its own granuloma (Fig. 1 B).

Macrophages also phagocytose Aβ. Highly aggregated Aβ accumulates within the vesicular compartments of microglia in mouse AD models (Baik et al., 2016; Spangenberg et al., 2019) or following injection of fluorescent fibrillar Aβ into the mouse brain (Fu et al., 2012). This aggregated Aβ, which can account for as much as 20% of the microglial cell volume (Huang et al., 2021; Spangenberg et al., 2019), is almost entirely concentrated in CD68^+^/LAMP1^+^ lysosomes (Condello et al., 2015; Fu et al., 2012; Spangenberg et al., 2019). Lysosomal localization and compaction is also seen when fibrillar Aβ is incubated with microglia in culture (Baik et al., 2016; Fu et al., 2012).

**Figure 2.**
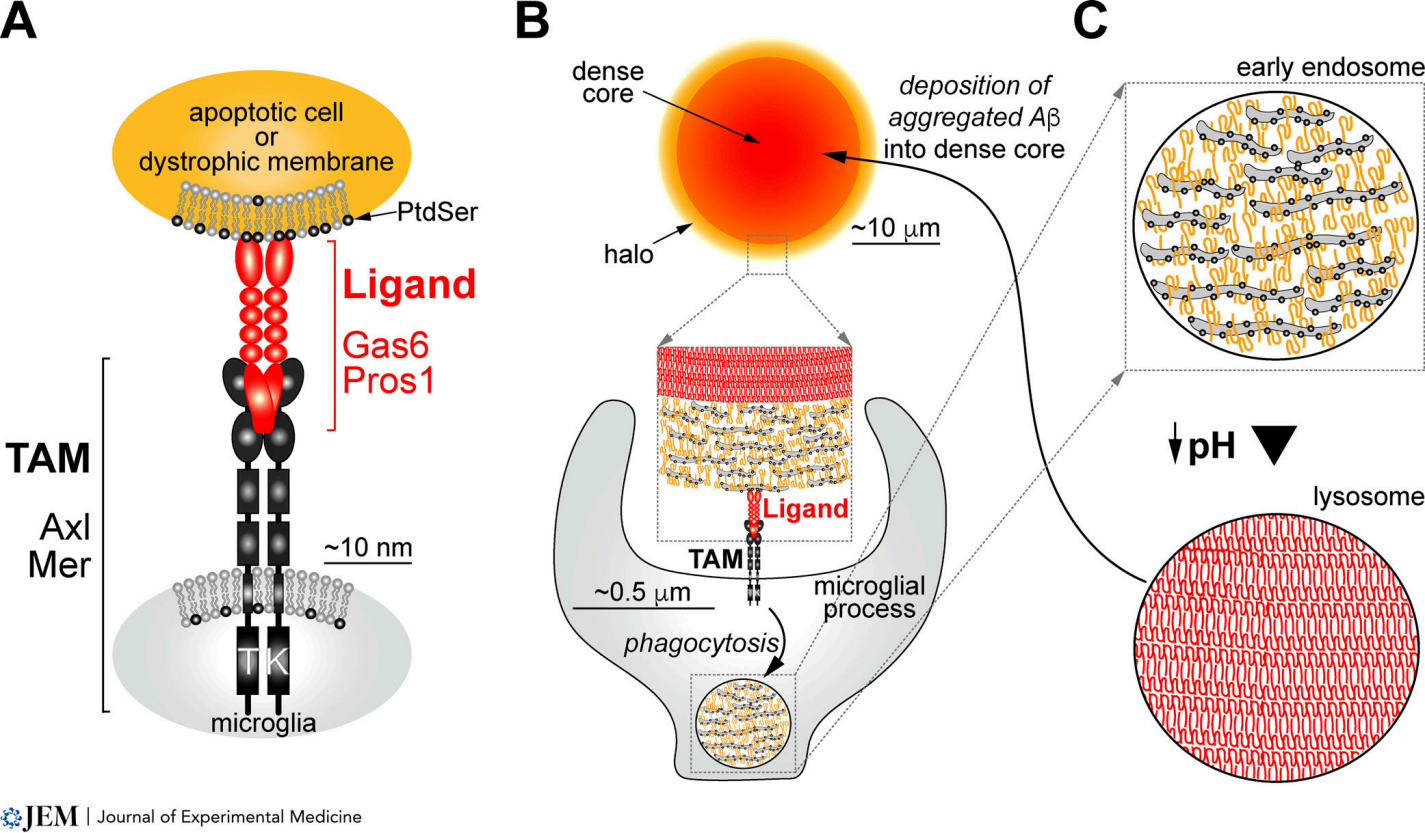
**Phagocytic construction of dense-core plaques via activation of microglial TAM receptors. (A)** The tripartite signaling arrangement assumed by the TAM receptor RTKs, their ligands, and PtdSer in all settings. In the brain, Mer and Axl are prominently expressed by activated microglia. The TAM ligands Gas6 and Pros1 are produced by many neural cells, including microglia and neurons. The critical phospholipid PtdSer is externalized on the surface of plasma membrane derived from cells damaged and/or killed by Aβ exposure. PtdSer, without which the kinase activity of TAM receptors cannot be effectively stimulated, binds to the amino terminus of Gas6 and Pros1. **(B)** Engagement of the microglial TAM system at the halo (yellow) of dystrophic PtdSer-rich membrane that contains Aβ oligomers and proto-fibrils. This halo surrounds the Thio S^+^ dense cores (red) that contain highly polymerized, aggregated, and compacted Aβ. TAM-mediated phagocytosis results in the internalization of Aβ oligomers and proto-fibrils into early microglial endosomes. **(C)** Aβ polymerization, aggregation, and compaction. Maturation of early microglial endosomes to lysosomes results in acidification of the organelle, which promotes Aβ aggregation. This aggregation is also promoted by the endosomal concentration of Aβ oligomers and proto-fibrils that is achieved by phagocytosis. Many Aβ-laden cells will die by apoptosis, and thereby deposit aggregated Aβ polymers into growing dense-core plaques. Some microglia may deliver aggregated Aβ to the cores of these plaques by exocytosis.

How is this Aβ internalized? As noted above, Aβ peptides polymerize into oligomers and proto-fibrils that are present in diffuse plaques that lack a dense core and also in the Aβ halos that surround dense-core plaques (Condello et al., 2015; Fig. 1C). In these settings, loosely organized Aβ is bound up with dystrophic plasma membrane derived from damaged and dead cells (Sharoar et al., 2019; Shi et al., 2017). This membrane turns out to be critical. It is indirectly recognized by two RTKs of the TAM family—Mer and Axl (Lemke, 2013; Lemke, 2019)—that play an essential role in the microglial phagocytosis of apoptotic cells (ACs; Fourgeaud et al., 2016; Fig. 2 A). In AC phagocytosis, the amino termini of the TAM ligands Gas6 and Protein S (Pros1) bind to phosphatidylserine (PtdSer), a membrane phospholipid that is displayed on the AC surface as an “eat-me” signal (Lemke, 2019). The carboxy termini of the TAM ligands concomitantly bind to Mer and Axl on microglia, activate the kinases of these receptors, and thereby initiate phagocytosis (Lemke, 2013; Fig. 2 A). This same arrangement is seen for microglial phagocytosis of Aβ. The dystrophic plasma membrane of diffuse plaques displays abundant externalized PtdSer, and all Aβ plaques are in turn decorated by Gas6 (and probably Pros1) that is bound to this PtdSer (Huang et al., 2021; Fig. 2 B). Finally, all microglia in contact with plaques express Mer and Axl. In vivo measurements demonstrate that the loss of TAM receptor signaling in the APP/PS1 mouse AD model reduces microglial Aβ phagocytosis 10-fold (Huang et al., 2021).

## Phagocytic construction of dense-core plaques via lysosomes

Microglial phagocytosis of Aβ has always been thought to constrain the growth of dense-core plaques (Long and Holtzman, 2019). Thus, a direct prediction of the dramatic phagocytic deficit that Mer/Axl-deficient microglia display is that TAM-deficient APP/PS1 brains should carry many more dense-core plaques than APP/PS1 mice with wild-type microglia. In reality, the TAM-deficient APP/PS1 mouse brain is populated with many fewer dense-core plaques (Huang et al., 2021).

What accounts for this result? The compaction of Aβ polymers and proto-fibrils into the β-pleated sheets of dense-core plaques has been shown to be driven by low pH (Su and Chang, 2001) and micromolar concentrations of Aβ oligomers (Tseng et al., 1999). Neither of these conditions is present in the extracellular space of the brain, but both are present in the confined acidic environment of lysosomes (Fu et al., 2012; Hu et al., 2009). As noted above, this is the compartment into which all phagocytosed Aβ is routed (Fig. 2, B and C). Once there, this internalized Aβ accumulates as an apparently indigestible mass. Clogged lysosomes are toxic to cells, and Aβ-laden mouse microglia undergo apoptosis (Baik et al., 2016). In addition, previously engulfed Aβ may be expelled by exocytosis (Arbo et al., 2020). Both of these events would allow for the deposition of aggregated, previously phagocytosed Aβ into the growing dense-core plaques of the AD brain (Fig. 2, B and C).

This hypothesis aligns with an established role for lysosomal dysfunction in AD (Nixon, 2017; Whyte et al., 2017). Genome-wide association studies have consistently linked genetic variation in lysosomal network genes to AD (Gao et al., 2018; Lambert et al., 2013), and many lysosomal storage diseases (Filocamo and Morrone, 2011), while clearly phenotypically distinct from AD, display neurodegenerative phenotypes (Platt et al., 2012). For example, mutations in the human *HEXB* gene, which encodes a subunit of the lysosomal enzyme β-hexosaminidase, cause Sandhoff disease, which is neurodegenerative (O’Dowd et al., 1986; O’Dowd et al., 1985), and *Hexb* mRNA is a microglial marker in the mouse (Masuda et al., 2020). *Hexb*^*−/−*^ mice and other lysosomal storage disease models exhibit amyloid-related histopathology (Annunziata et al., 2013; Keilani et al., 2012), and it has recently been shown that *Hexb*^*+/−*^ heterozygotes, when crossed into the amyloidogenic App^NL-G-F^ mouse (Saito et al., 2014), exhibit decreased Aβ deposition relative to App^NL-G-F^ alone (Whyte et al., 2022). All of these observations are consistent with the aggregation and compaction of internalized Aβ in microglial lysosomes (Fig. 2, B and C).

While the density of Thio S^+^ dense-core plaques is reduced in TAM-deficient APP/PS1 mice, three markers of AD pathology are concomitantly increased (Huang et al., 2021). The first is the prevalence of diffuse, poorly organized plaques. The second is the size of the halos of Aβ oligomer-rich dystrophic membrane that surround plaques, which are labeled by the endolysosomal marker LAMP1 and the ER marker reticulon-3. The third is the deposition of diffuse Aβ within and around the walls of blood vessels. This deposition (cerebral amyloid angiopathy) is also increased in TAM-deficient APP/PS1 mice, just as it is in microglia-depleted 5xFAD mice (Spangenberg et al., 2019).

Some features of microglial interaction with plaques in TAM-deficient APP/PS1 mice resemble those seen when mice lacking the microglial phagocytic receptor Trem2 (Ulland and Colonna, 2018; Wang et al., 2015) are crossed with amyloidogenic mouse AD models (Huang et al. [2021] and references therein). Genetic variants in the human *TREM2* gene have been found to increase the risk of developing late-onset AD (Ulland and Colonna, 2018), and plaque–microglial interactions have been studied in human AD patients carrying the most widely studied of these *TREM2* variants, designated R47H (Yuan et al., 2016). These analyses revealed that the incidence of dense-core plaques was modestly reduced in R47H patients, while the incidence of poorly organized (“filamentous”) plaques was increased (Yuan et al., 2016), mirroring the changes in these plaques seen in TAM-deficient APP/PS1 mice (Huang et al., 2021). At the same time, microglial binding to plaques in R47H patients was decreased in parallel with the decreased binding seen in TAM-deficient APP/PS1 mice (Huang et al., 2021; Yuan et al., 2016). Together, all of the above data support the hypothesis that microglial phagocytosis of Aβ material does not destroy dense-core plaques as heretofore imagined. Exactly to the contrary, it creates these plaques.

## The transcriptomic response to bacterial versus peptide pathogens

**Figure 3.**
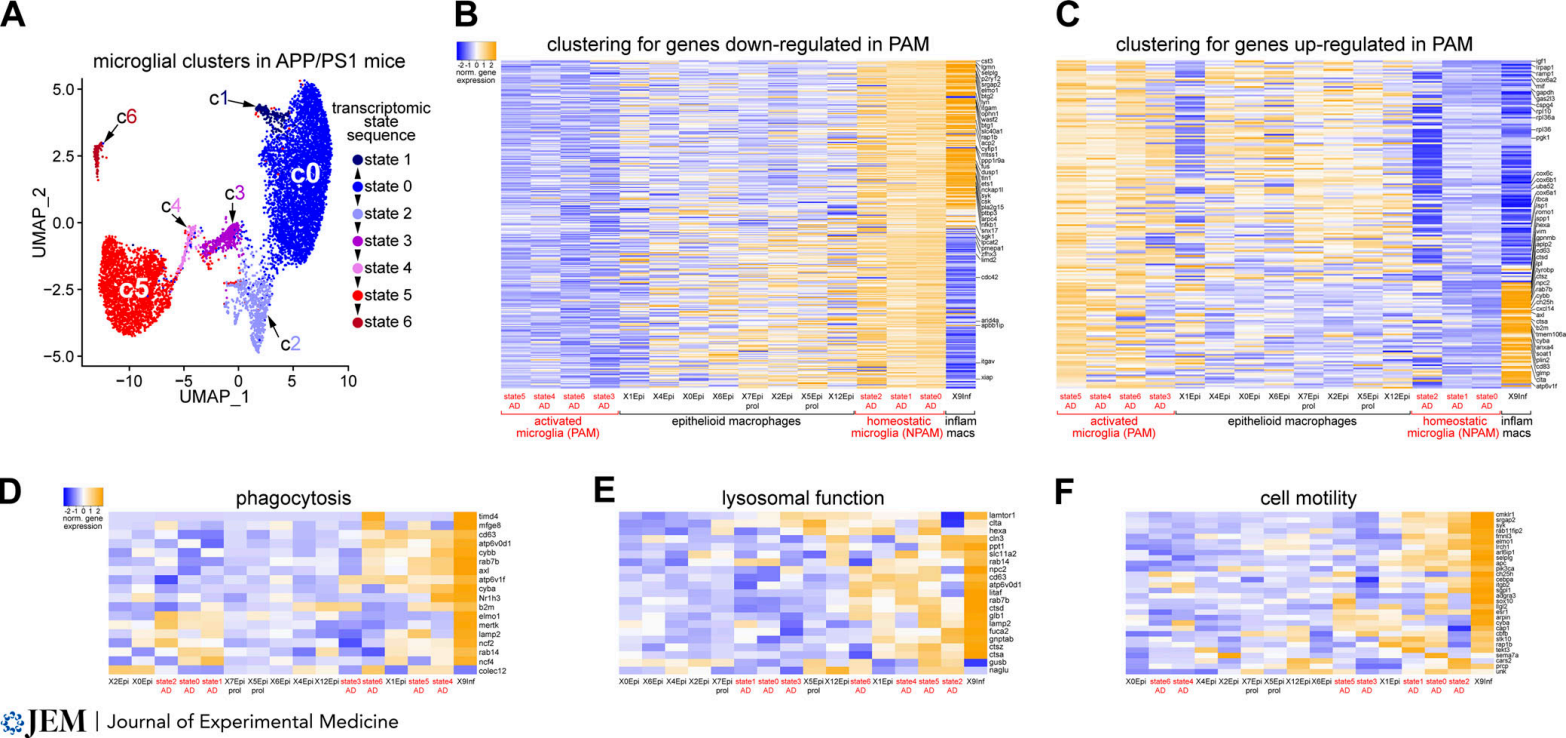
**The transcriptomic response of PAM versus *M. marinum*–associated macrophages. (A)** Seven microglial cell clusters (c0–6) defining seven transcriptomic states in APP/PS1 Alzheimer’s mice, generated by Seurat analysis of single-cell RNA-seq results and displayed using uniform manifold approximation and projection (UMAP). Clusters 0 and 5 are the principal homeostatic (NPAM) and activated (PAM) clusters, respectively. Modified from Huang et al. (2021), with permission. **(B)** Comparative composite heatmaps for the indicated genes (mRNAs) across microglial transcriptomic clusters in APP/PS1 mice (as in A) and macrophage transcriptomic clusters in the *M. marinum* granuloma (as in Cronan et al. [2021]). X0Epi, X1Epi, etc. are epithelioid macrophages clusters, and X9Inf is the inflammatory macrophage cluster (Cronan et al., 2021). The differentially expressed genes shown are down-regulated in microglial state 5 (PAM) versus state 0 (NPAM), with the criteria of log(fold-change) >0.25 and adjusted P value <0.05. Columns are organized by similarity, based on unsupervised hierarchical clustering using the heatmap.2 function in R. Values shown as z-scaled log-transformed normalized average gene expression for each group. **(C)** The differentially expressed genes shown are up-regulated in microglial state 5 (PAM) versus state 0 (NPAM). Columns are organized by similarity, based on clustering. **(D–F)** Hierarchical clustered normalized expression of the indicated phagocytosis genes (D), lysosomal function genes (E), and cell motility genes (F) across microglial transcriptomic clusters in APP/PS1 mice and macrophage transcriptomic clusters in the *M. marinum* granuloma.

Mycobacterial granulomas and dense-core Aβ plaques have different triggers and are assembled by qualitatively different macrophages in different tissues (Bohlen et al., 2019; Liegeois et al., 2018). Are there any commonalities with respect to the macrophage transcriptomic responses in such divergent settings? Methodologically comparable 10x Genomics single-cell RNA-sequencing (RNA-seq) analyses have been performed for both granuloma macrophages from zebrafish infected with *Mycobacterium marinum* (Cronan et al., 2021), a widely studied fish model for *M. tuberculous* infection (Ramakrishnan, 2020), and microglia associated with dense-core Aβ plaques in the APP/PS1 mouse model of AD (Huang et al., 2021). In contrast to the infection of mouse lungs with *M. tuberculosis*, which yields noncaseating and nonnecrotic lesions dominated by neutrophils, the granulomas that form in fish after infection with *M. marinum* more closely resemble the pulmonary granulomas seen in TB patients (Cronan et al., 2021). We therefore performed a systematic comparative meta-analysis of the zebrafish macrophage and mouse microglia data sets (Fig. 3).

Of the genes analyzed in the two studies, we identified 7,710 homologous genes whose mRNAs were the basis for our comparison. Seurat-based analyses generated nine macrophage clusters in the fish. Essentially all of these corresponded to cells that were to some extent activated, as cells were isolated from dissected granulomas. Eight clusters represented epithelialized (epithelioid) macrophages (X1Epi, X2Epi, etc. in Fig. 3), while a single cluster was populated by inflammatory macrophages that are thought to reside adjacent to the necrotic granuloma core (Cronan et al., 2021; XInf in Fig. 3). The microglia analyzed in plaque-burdened APP/PS1 mice were CD45^+^ cells dissociated from the cortex, and so represented both activated plaque-associated (PAM) and homeostatic non–plaque-associated (NPAM) microglia. Seurat analyses generated seven microglial clusters, which were progressively arrayed between homeostatic (clusters 0, 1, and 2) and activated (clusters 4, 5, and 6) states (Huang et al., 2021; Fig. 3 A).

We performed an unsupervised hierarchical ordering of clusters with respect to genes down-regulated in PAM compared with NPAM. This ordering revealed that epithelioid macrophages in the *M. marinum* granuloma, while intermediate in activation between these states, more closely resembled PAM than NPAM (Fig. 3 B). Zebrafish homologs of down-regulated PAM mRNAs that were also down-regulated in epithelioid macrophage clusters included the selectin P ligand *selpg*, the integrin αM *itgam*, the ferroportin *slc40a1*, and the transcription factor *nfkb1* (Fig. 3 B). An equivalent ordering of mRNAs up-regulated in PAM versus NPAM showed that the homologous zebrafish mRNAs of epithelioid macrophages again more closely resembled PAM (Fig. 3 C). Representative mRNAs elevated in both PAM and epithelioid macrophages included the inflammatory regulator *ramp1*, the proteoglycan *csp4* (*ng2*), the cytochrome c oxidase subunit *cox6c*, and the tubulin chaperone *tbca* (Fig. 3 C).

The inflammatory macrophages of the zebrafish granuloma exhibited a bifurcated profile and contained mRNAs whose normalized average expression was strongly concordant with either mouse PAM or NPAM in regulation (Fig. 3, A and B). Consistent with the biology discussed above, mRNAs associated with phagocytosis, including the phagocytic receptors *timd4* and *axl*, the phagocytic ligand *mfge8*, and the endosomal GTPase *rab7b*, were up-regulated in inflammatory macrophages of the granuloma and also in PAM (Fig. 3 D). Similarly PAM-concordant up-regulation was seen for mRNAs tied to lysosomal function, including the intracellular cholesterol transporter *npc2*, the tetraspanin *cd63*, the lysosomal protease *ctsd*, and the GlcNac phosphotransferase *gnptab* (Fig. 3 E). In contrast, NPAM-concordant up-regulation in granuloma inflammatory macrophages was seen for many mRNAs tied to cell motility, including the migration regulator *srgap2*, the cytoplasmic tyrosine kinase *syk*, and the directed cell migration regulator *apc* (Fig. 3 F). The expression of nearly all of these motility regulators was also lower in most epithelioid macrophages. This coregulation in macrophages of the granuloma and NPAM of the AD brain is consistent with the observations that (a) PAM and their processes are less motile than NPAM in the AD brain, and (b) epithelioid macrophages are essentially immobile by virtue of being interdigitated.

Single nucleus RNA-seq analyses have also been performed with nuclei isolated from frozen postmortem brain tissues of human AD patients (Grubman et al., 2019; Mathys et al., 2019; Morabito et al., 2021; Zhou et al., 2020). Although the ability of these data sets to fully capture microglial activation in human AD has been questioned (Thrupp et al., 2020), we nonetheless detected many correspondences when they were compared to the mRNAs that are coordinately regulated between mouse PAM and zebrafish inflammatory macrophages. Among the mRNAs that were similarly up-regulated in human microglia in at least one single nucleus RNA-seq data set are those encoding the lysosomal hexosaminidase *HEXA*, the osteopontin *SPP1*, the cathepsin protease *CTSD*, the vacuolar sorting protein *VPS13C*, the plexin-related protein *PLXDC2*, and the Trem2 adaptor protein *TYROBP*. Similarly down-regulated were mRNAs encoding the tropomyosin *TPM3*, the endocytic protein *NUMB*, the sialomucin *CD164*, and the acyltransferase *LPCAT2*.

Single-cell transcriptomic data have also recently been published for macrophages collected from the pulmonary granulomas that develop when nonhuman primates are infected with *M. tuberculosis* (Gideon et al., 2022). Again, many granuloma macrophage mRNAs from this macaque model were also coregulated in both mouse PAM and the inflammatory macrophages of zebrafish granulomas. Among these mRNAs are those encoding the cholesterol transporter *NPC2* (discussed above), the lipoprotein lipase *LPL*, the GTPase *RAB7B* (also discussed above), *AXL*, the lysosomal ATPase *ATP6V1F*, and again, *TYROBP*.

Finally, single-cell RNA-seq analyses of Aβ plaque–associated human microglia have linked these cells to a critical population of macrophages that populate an entirely different “plaque” in human vascular disease—namely the atherosclerotic plaques of atherosclerosis (Claes et al., 2021). In those studies, microglia differentiated from human induced pluripotent stem cells prepared from both normal and TREM2R47H patients were xenografted into an amyloidogenic mouse AD model and analyzed 7 mo after transplantation, when Aβ plaques in the brain were abundant. It was observed that the PAM in these xenografts displayed a transcriptomic profile with remarkable resemblance to that of foam cells, the lipid-laden macrophages of human atherosclerotic plaques, with high expression of *SPP1*, *CTSD*, *NPC2*, and many of the other mRNAs discussed above (Claes et al., 2021). This is an especially interesting observation, since (a) foam cells, or “foamy macrophages,” are also components of the TB granuloma (Russell et al., 2009; Shim et al., 2020; Fig. 1 B); (b) macrophage phagocytosis and expression of the phagocytic receptor Mer are critical to the formation and structural integrity of atherosclerotic plaques (Tao et al., 2020); (c) lipid droplet–accumulating microglia are increasingly abundant in aging mouse and human brains and are prominent in amyloidogenic mouse models (Marschallinger et al., 2020); and (d) cholesterol deposition, the defining histopathological feature of atherosclerosis, is also seen in the dense-core Aβ plaques of AD (Di Paolo and Kim, 2011; Mori et al., 2001). Together, all of the above observations indicate that the macrophages populating *M. marinum* and *M. tuberculosis* granulomas in fish and monkeys, together with the foam cell macrophages populating human atherosclerotic plaques, share many of the transcriptomic responses exhibited by the microglia that are bound to the dense-core Aβ plaques of AD in mice and humans.

## Implications of the granuloma hypothesis

If dense-core Aβ plaques are granulomas, how should they be addressed in the context of AD therapy? This is an important question, since these plaques are often detected in cognitively healthy adults (Bennett et al., 2006; Jagust, 2016). Perhaps the most salient prediction of the granuloma hypothesis is that agents that break up dense-core plaques—but that do not concomitantly reduce the production and accumulation of Aβ peptides, oligomers, and proto-fibrils, or alternatively stimulate the efflux of these components from the brain—are likely to be of limited value as AD therapeutics. While dense-core plaques are obviously not beneficial with respect to brain function, they may be less damaging to the brain than widely disseminated Aβ oligomers and proto-fibrils. As noted above, most of these lower-order polymers are neurotoxic. We suggest that microglial formation of dense-core plaques may be an attempt to make the best of a bad situation—the overproduction and accumulation of Aβ peptides.

The first drug approved by the US Food and Drug Administration (FDA) to address the underlying biology of AD is aducanumab, a monoclonal antibody against Aβ (Sevigny et al., 2016). Extended treatment with aducanumab demonstrated only marginal benefit with respect to the improvement of patient cognitive outcomes, and this was seen at only high doses in only one of two large clinical trials (Rabinovici, 2021). These results led an FDA advisory panel to vote overwhelmingly against drug approval. In a controversial decision (Mullard, 2021), FDA leadership nonetheless approved aducanumab, now sold as Aduhelm. The principal justification given for this decision was not improved clinical outcomes, but rather the unambiguous ability of aducanumab to substantially reduce patient expression of a surrogate biomarker over time. This surrogate biomarker was amyloid burden in the brain, as assessed by PET imaging with the radiopharmaceutical florbetapir (^18^F; Jaffe, 2021; Sevigny et al., 2016). Florbetapir (Choi et al., 2009; Zeng and Goodman, 2013) is a compound that has been shown to bind almost exclusively to Thio S–labeled plaques on postmortem brain sections from AD patients (Choi et al., 2012). As noted above, Thio S intensely stains dense-core plaques but only weakly binds to diffuse plaques, so the principal amyloid structures identified by florbetapir PET in AD patients are almost certainly classic dense-core plaques. It is possible that aducanumab may have additional effects beyond dense-core plaque reduction, but for the most part these have not been rigorously assessed in patients. The US Center for Medicare and Medicaid Services recently considered all of the above findings and announced that it will not pay for Aduhelm outside of its use in a controlled clinical trial.

Although the initiating event with respect to amyloid deposition in AD is the excess production of Aβ peptides, multiple pharmacological inhibitors of BACE1 and γ-secretase—the enzymes that sequentially cleave these peptides from APP—have failed as AD therapies, often because of adverse off-target effects (Coric et al., 2015; Das and Yan, 2019). Compounds that allosterically modulate, rather than inhibit, γ-secretase activity may represent promising new approaches to reducing Aβ peptide levels (Rynearson et al., 2021). Based on the concepts delineated in this Perspective, additional alternative AD therapies that might be considered going forward include agents that promote microglial phagocytosis of loosely organized Aβ. The orally available PPARγ agonist pioglitazone stimulates both macrophage expression of Mer and Mer-dependent microglial phagocytosis (Savage et al., 2015). This transcription factor agonist, which is already FDA-approved for type 2 diabetes and other indications, ameliorates symptoms in mouse AD models (Savage et al., 2015) but has not shown therapeutic efficacy in clinical trials in AD. It may therefore be useful to screen for more Mer-selective and brain-penetrant stimulators of microglial Mer expression or kinase activity as potential next-generation therapeutics. Similarly useful would be screens for stimulators of additional non-TAM phagocytic pathways that operate in microglia. At the very least, the advisability of simple dense-core plaque disaggregation as an AD therapy should be reevaluated.
